# Turn-of-the-candle effect in bitcoin returns

**DOI:** 10.1016/j.heliyon.2023.e14236

**Published:** 2023-03-02

**Authors:** Savva Shanaev, Mikhail Vasenin, Roman Stepanov

**Affiliations:** University of Northumbria at Newcastle, United Kingdom, NE8 1ST, UK

**Keywords:** Bitcoin, Cryptocurrency, High frequency, Market efficiency, Market anomaly

## Abstract

This study discovers a statistically and economically significant intraday anomaly on Bitcoin markets. Positive returns of 0.58 bps per minute are disproportionately concentrated at the turn of 15-min candles (in minutes 0, 15, 30, and 45 of each trading hour). Average returns in other trading minutes are negative. The effect is consistent across Bitcoin exchanges, in quantile regression models, and TGARCH-M estimations with heavy tails, and persist in out-of-sample tests. A high-frequency strategy that exploits this “turn-of-the-candle” effect can be net-outperforming with initial investment as low as $5,000. The anomaly is detected in the data starting from mid-to-late 2020, is potentially associated with algorithmic trading relying on the arrival of 15-min candle information, and its discovery contributes significantly to the understanding of cryptocurrency adaptive market efficiency.

## Highlights

• Bitcoin returns are substantially higher at the turn of 15-min candles.
• The effect has originated mid-to-late 2020 on all seven sample exchanges.
• Results are robust to outliers, TGARCH-M effects, and heavy tails.
• An anomaly-exploiting strategy outperforms buy-and-hold after fees and spreads.
• The effect can be associated with high-frequency algorithmic trading.

## 1 Introduction

Since the introduction of Bitcoin in 2009 as the first cryptocurrency, a voluminous body of empirical research has been developed seeking to investigate its financial properties as an alternative investment. It has long been established that the Bitcoin market is weak-form inefficient with patterns more consistent with efficiency on daily data being reported recently [[5], [25],^34^,^35^]. However, little to no conventional well-established calendar anomalies or seasonal patterns have thus far been discovered in daily Bitcoin returns [16,21,28], with prior studies asserting that seasonality in cryptocurrency follows unconventional patterns different to those documented on well-established financial markets [9,31] or that such seasonality is time-varying and dynamic [18]. While the broader market efficiency literature shifted towards the use of intraday high-frequency data [7,30,32] or, on the contrary, shifted towards long memory and long-term dependence research that utilises more advanced techniques such as fractal analysis [3,23,24] or flexible Fourier forms [2], more specific seasonality research on such samples remains extremely scarce.

This study addresses this gap in the literature by discovering a recent (appearing mid-to-late 2020), and well-defined intraday “turn-of-the-candle” anomaly in Bitcoin markets. It shows that positive returns are disproportionately concentrated at 0^th^, 15^th^, 30^th^, and 45^th^ minutes of each trading hour, corresponding to the turns of 15-min candles that are closely followed by high-frequency traders. This effect is statistically (a t-stat above nine for all seven sample exchanges in 2021) and economically (an anomaly-exploiting strategy net-outperforms buy-and-hold after fees and bid-ask spreads with starting capital as low as $5,000) significant. Moreover, the results are robust across exchanges, to outliers and heavy tails. Therefore, this research contributes to the literature on intraday cryptocurrency price dynamics, cryptocurrency market efficiency, market anomalies, and high-frequency trading strategies.

The rest of this paper is organised as follows: the next section briefly overviews the literature on intraday cryptocurrency market dynamics. Further, the sources of data and the estimation strategy used by the study are discussed. The findings section presents the empirical results and the necessary robustness checks. The final section concludes with key theoretical and practical implications.

## 2 Intraday bitcoin price dynamics

Early research on the intraday dynamics of Bitcoin utilised primarily hourly or half-hourly data. As such, Hu et al. [15] investigate intraday behaviour of major cryptocurrencies and find price clustering at round numbers for hourly frequency. Urquhart and Zhang [36] apply the DCC-GARCH framework to hourly Bitcoin and conventional currency returns, investigating cryptocurrency hedging and safe haven properties. Shen et al. [32] utilise half-hourly data from major Bitcoin exchanges to document intraday momentum trading profitability, with abnormal returns of such strategies remaining positive after accounting for fees, especially for leveraged investors. Chu et al. [11] provide evidence of evolving cryptocurrency market efficiency in line with the adaptive market hypothesis on hourly basis and argue that higher-frequency data is required to investigate the return dynamics of Bitcoin. This supports earlier findings of Khuntia and Pattanayak [17] on daily data who stress that research on high-frequency trading strategies should be quantitatively rigorous and consider their time-varying profitability as well as transaction costs.

However, the highest data frequency utilised by an absolute majority of contemporary studies is 5-min candles. The earliest piece of empirical work in this area is Pichl and Kaizoji [27] who investigate Bitcoin volatility on 5-min, hourly, and daily basis. The authors use a neural network model to predict Bitcoin’s log-returns. Eross et al. [13] report increasing intraday returns as well as time-varying volatility and liquidity of Bitcoin trading on a 5-min frequency, with liquidity being highest during opening times of conventional financial markets. Similarly, Petukhina et al. [26] exploit 5-min pricing data to reinforce the dependence of Bitcoin market activity on the daily human activity cycle and global financial markets, thus arguing that algorithmic trading was not material on cryptocurrency exchanges as of mid-2018. Most recently, Bouri et al. [7] utilise cumulative intraday abnormal returns on 5-min frequency to establish an intraday trading strategy which net-outperforms the buy-and-hold strategy on Bitstamp when the transaction fee structure is accounted for. The importance of fees and bid-ask spreads in high-frequency cryptocurrency trading has been also highlighted by Dyhrberg et al. [12] and Scharnowski [29] who emphasise that fees and spreads in Bitcoin trading are lower than on equity markets and importantly are decreasing as the market matures, evidencing retail and institutional investability of Bitcoin. Furthermore, Wei [37] and Brauneis and Mestel [8] establish a positive relationship between liquidity and efficiency in cryptocurrencies, implying anomalies on Bitcoin markets could be driven by limits to arbitrage and are thus not associated with net abnormal returns. Potentially supporting this claim, existing research on profitable intraday trading strategies in cryptocurrencies mostly proposes algorithmically and computationally complex techniques [7,22,32].

Hence, the primary objective of this study is to address the identified gap in the literature, utilising 1-min candles to investigate intraday Bitcoin seasonality and establish a conceptually simple net-profitable anomaly-exploiting strategy, while acknowledging the adaptive market hypothesis [11,17], trading fee structure, bid-ask spreads, potential heterogeneities across Bitcoin exchanges [[7], [10],^12^,^29^], as well as heavy tails and the prominence of outliers in Bitcoin returns [14,21].

## 3 Data and methodology

This study collects the Bitcoin price from seven exchanges which allow the download of 1-min candles using public API. The sample starts on the first day of the first available month when a particular exchange provided minutely price quotes (see Table 1 below) and is cut off on December 31, 2021. Exchange-provided data is used instead of aggregated sources such as coinmarketcap.com to study the trading implications of the anomaly more robustly and given data quality considerations emphasised by prior literature [1]. The data on exchange fee structure is collected from their respective websites to implement investment simulations, following [7]. All seven sample exchanges provide fee discounts depending on 30-day rolling trading account volume, and all except Binance offer zero fees to clients with sufficiently large volumes. The lowest threshold is provided by Bitfinex (7,500,000 USD). Therefore, Bitfinex is chosen to execute the trading strategy simulations to test for robustness and economic significance of the results for retail investors. Following prior literature [29], bid-ask spreads for Bitfinex are collected from data.bitcoinity.org.

**Table 1:** Sample exchanges.

Exchange	Data available from	New account fee, basis points	Zero fee 30-day volume threshold
Bitfinex	April 01, 2013	10	7,500,000 USD
Bittrex	June 01, 2018	35	60,000,000 USD
Binance	August 01, 2017	10	None
Gemini	October 01, 2015	25	15,000,000 USD
Kucoin	January 01, 2019	10	2,000 BTC
Bitstamp	January 01, 2017	50	20,000,000,000 USD
FTX	January 01, 2020	2	25,000,000 USD

The performance of the simulated traded strategy is further evaluated using the probabilistic Sharpe ratio framework as in Bailey and Lopez de Prado [4] to illuminate the statistical and economical significance of outperformance and address data-snooping concerns while also notably accounting for heavy tails of the Bitcoin return distribution.

(1) $$PSR=\Phi \left(\frac{\hat{SR}-SR^{*}}{\sqrt{\left(1-S*\hat{SR}+\frac{K+2}{4}*{\hat{SR}}^{2}\right)/\left(n-1\right)}}\right)$$

In equation (1) above, PSR is the probabilistic Sharpe ratio interpretable as a probability of the estimated strategy Sharpe ratio $\hat{SR}$ exceeding the target Sharpe ratio $SR^{*}$ of the buy-and-hold strategy, S and K are the skewness and kurtosis of the strategy return distribution, n is the number of sample days, and Φ is the cumulative normal distribution function.

Next, the study estimates the regression model with dummy variables:

(2) $$r_{it}=c_{i}+\kappa_{i}D_{t}+\varepsilon_{it}$$

Where $r_{it}$ is the 1-min log-return on the i th exchange in minute t; $c_{i}$ is the constant; $D_{t}$ is a dummy variable equal to one if minute t is at the turn of the 15-min candle (minutes 0, 15, 30, or 45), and zero otherwise; $\kappa_{i}$ is the estimator of the turn-of-the-candle effect in the exchange i; and $\varepsilon_{it}$ is the error term. Equation (2) is estimated using heteroskedasticity and autocorrelation consistent standard errors in 1) conventional OLS and 2) quantile regression form [19] to account for the impact of outliers and heavy tails prominent on Bitcoin markets [14,[21], [33]]. To determine the timing of the anomaly’s origin, 1) the equation is estimated across sample years, and 2) a Chow structural shift test is additionally undertaken for the $\kappa_{i}$ estimator across sample months m.

(3) $$r_{it}=c_{i}+\kappa_{i}D_{t}+\delta_{mi}M_{mt}+\varepsilon_{it}$$

In equation (3) above, $\delta_{mi}$ is the differential turn-of-the-candle effect on the i th exchange for a structural break in month m, and $M_{mt}$ is a dummy variable equal to one if minute t occurs within or after sample month m and zero otherwise.

Further, a TGARCH-M model [6,38] with Gaussian, Student’s t, and generalised error distribution as well as variance- and volatility-based risk premia is employed to simultaneously control for explosive behaviour and potential risk-related explanations of the effect:

(4) $$r_{t}=c+\mu \sigma_{t-1}^{2}+\kappa D_{t}+\varepsilon_{t}\left(0,\sigma_{t}^{2}\right)$$

$$\sigma_{t}^{2}=\omega +\alpha \varepsilon_{t-1}^{2}+\beta \sigma_{t-1}^{2}+\theta \varepsilon_{t-1}^{2}I\left(\varepsilon_{t-1}<0\right)$$

In equation (4) above, $r_{t}$ is the 1-min log-return in minute t; c is the constant; $D_{t}$ is the turn-of-the-candle dummy; κ is the turn-of-the-candle effect estimator; μ is the GARCH-M conditional variance (or conditional volatility) risk premium; $\varepsilon_{t}$ is the error term following a normal distribution, a Student’s t distribution, or a generalised error (GED) distribution depending on the estimation with conditional variance $\sigma_{t}^{2}$. Variance dynamics is modelled using a TGARCH process, with ω, α, β, and θ denoting unconditional variance, immediate disturbance, conditional variance persistence, and the leverage effect, respectively. Shape parameters for Student’s t and GED distributions are allowed to vary to maximise the log-likelihood function. The use of a battery of GARCH models for robustness is essential given the prominence of conditional heteroskedasticity effects in cryptocurrencies and notable heterogeneity across various coins in terms of the best-fitting GARCH model classes [20].

Finally, this study executes an out-of-sample test, evaluating the turn-of-the-candle effect on Bitfinex in January–August 2022. Advanced estimations and trading strategy simulations are performed on Bitfinex data because of the maturity and liquidity of the exchange as well as its attractiveness for higher-frequency trading due to lower fees and bid-ask spreads [29]. Data and code for the estimations are available upon request.

## 4 Findings and discussion

Table 2 below reports the baseline estimations for the turn-of-the-candle effect. The effect is consistently significant across the full sample for all seven exchanges, although the magnitude of the effect is highest in 2021 (all t-statistics above nine). The turn-of-the-candle anomaly is not documented in the earlier years (2013–2016) and first emerges on Bitfinex and Gemini in 2017, although no similar effects exist on Binance and Bitstamp (the other two sample exchanges operating in 2017). The emergence of the anomaly in 2017 and 2020 could potentially suggest a bullish cryptocurrency market sentiment explanation. However, the effects are most consistent across exchanges in 2021 (0.82–0.97 basis points per minute).

**Table 2:** The turn-of-the candle effect on major Bitcoin exchanges.

Year	Exchange						
	Bitfinex	Bittrex	Binance	Gemini	Kucoin	Bitstamp	FTX
2013	−0.7191						
	(1.9535)						
	*0.7128*						
2014	0.4148						
	(0.2758)						
	*0.1327*						
2015	−0.0589			−0.4638			
	(0.1000)			(0.4079)			
	*0.5555*			*0.2555*			
2016	0.1100			0.0129			
	(0.1495)			(0.0365)			
	*0.4619*			*0.7224*			
2017	0.2139**		−0.0211	0.5227***		−0.0847	
	(0.0886)		(0.2587)	(0.0768)		(0.0986)	
	*0.0158*		*0.9349*	*0.0000*		*0.3902*	
2018	0.1732**	0.1422	0.2618***	0.0751		0.1149	
	(0.0803)	(0.0890)	(0.0849)	(0.0778)		(0.0818)	
	*0.0310*	*0.1102*	*0.0020*	*0.3343*		*0.1603*	
2019	0.1392**	0.0521	0.1741***	0.0646	0.1703***	0.1074	
	(0.0594)	(0.0614)	(0.0611)	(0.0638)	(0.0637)	(0.0665)	
	*0.0191*	*0.3960*	*0.0044*	*0.3113*	*0.0075*	*0.1061*	
2020	0.3027***	0.3208***	0.3330***	0.3851***	0.3277***	0.3133***	0.2791***
	(0.0700)	(0.0702)	(0.0731)	(0.0794)	(0.0704)	(0.0787)	(0.0704)
	*0.0000*	*0.0000*	*0.0000*	*0.0000*	*0.0000*	*0.0000*	*0.0000*
2021	0.9024***	0.8186***	0.9008***	0.9679***	0.8841***	0.9282***	0.8745***
	(0.0855)	(0.0841)	(0.0896)	(0.0879)	(0.0876)	(0.0887)	(0.0870)
	*0.0000*	*0.0000*	*0.0000*	*0.0000*	*0.0000*	*0.0000*	*0.0000*
Full sample	0.2500***	0.3555***	0.3804***	0.3131***	0.4667***	0.2753***	0.5798***
	(0.0868)	(0.0379)	(0.0419)	(0.0324)	(0.0433)	(0.0374)	(0.0561)
	*0.0040*	*0.0000*	*0.0000*	*0.0000*	*0.0000*	*0.0000*	*0.0000*

**Notes:** Robust standard errors reported in parentheses and p-values presented in italics. ***, **, and * denote statistical significance at 1%, 5%, and 10%, respectively.

**Fig. 1:**
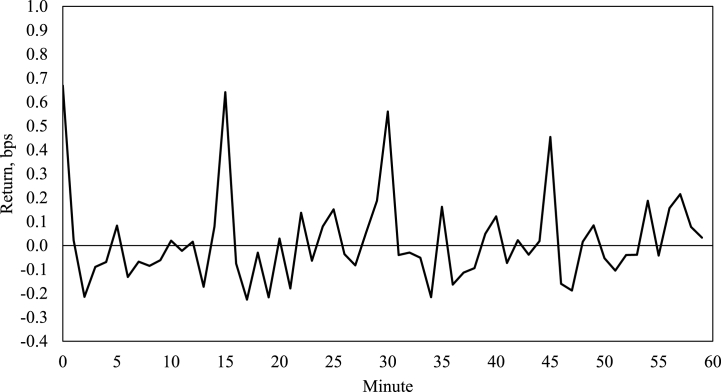
The example of turn-of-the-candle effect (Bitfinex, 2021).

Fig. 1 below visualises average 1-min returns across candles on Bitfinex in 2021. The turn-of-the-candle returns (0^th^, 15^th^, 30^th^, and 45^th^ minutes) are discernibly the highest and generate average gain of 0.58 bps per minute (from 0.55 bps on Bittrex to 0.66 bps on Gemini), while the average return for all other minutely candles across all exchanges is *negative*. There is little evidence of any 10-min seasonal cycles on Bitcoin returns, therefore the effect must be associated with the trading patterns or broader financial properties of the cryptocurrency rather than its blockchain fundamentals (with a block being mined every 10 min on average, and the anomaly emerging in 15-min patterns).

**Fig. 2:**
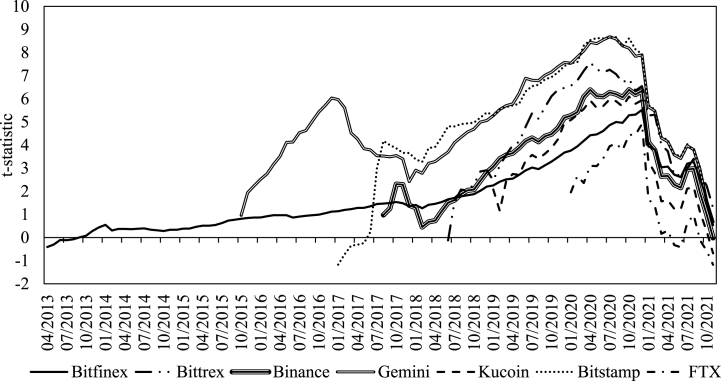
The timing of the turn-of-the candle anomaly on major Bitcoin exchanges.

Fig. 2 below reports monthly Chow structural shift t-statistics for the turn-of-the-candle effect. It is evident that the anomaly has originated in mid-to-late 2020, at first on Bittrex (April 2020), then on Gemini and Bitstamp (July 2020), Binance and Kucoin (October 2020), and finally on Bitfinex and FTX (December 2020). This largely contradicts the speculation that the effect is associated with positive investor sentiment, as the late-2020 Bitcoin bullish run started in November. However, the effect reinforces the need to perform additional robustness checks on 2021 data when the anomaly has already emerged on all exchanges.

Table 3, Table 4 below present the output of such robustness tests. Quantile regression conditional median models demonstrate that the turn-of-the-candle effect in 2021 cannot be fully attributed to outliers or extreme positive returns, while TGARCH-M estimations show the anomaly is robust to autoregressive conditional heteroskedasticity, heavy tails in Student’s t and generalised error distributions, and time-varying risk-pricing. The shape parameter for Student’s t probability density function is higher than four, implying that the minutely Bitcoin return distribution has finite variance, well-defined skewness and kurtosis, yielding potentially attractive investment properties of an anomaly-exploiting strategy for a wide range of individual and institutional investors.

**Table 3:** Robustness check: quantile regression conditional median estimations (2021).

Year	Exchange						
	Bitfinex	Bittrex	Binance	Gemini	Kucoin	Bitstamp	FTX
Conditional median (2021)	0.3943***	0.0252*	0.3666***	0.4672***	0.7103***	0.6761***	0.6470***
	(0.0267)	(0.0150)	(0.0222)	(0.0322)	(0.0454)	(0.0421)	(0.0467)
	*0.0000*	*0.0927*	*0.0000*	*0.0000*	*0.0000*	*0.0000*	*0.0000*

**Notes:** Robust standard errors reported in parentheses and p-values presented in italics. *** and * denote statistical significance at 1% and 10%, respectively.

**Table 4:** Robustness check: TGARCH-M model results (Bitfinex, 2021).

TGARCH-M	Turn-of-the-candle	ω	Α	β	θ	μ	ν
Gaussian tails; volatility-based risk pricing	0.4932***	0.9743***	0.0690***	0.9176***	0.0221***	0.0215***	
	(0.0307)	(0.0048)	(0.0002)	(0.0001)	(0.0004)	(0.0035)	2.0000
	*0.0000*	*0.0000*	*0.0000*	*0.0000*	*0.0000*	*0.0000*	
Student’s t tails; volatility-based risk pricing	0.4367***	0.4424***	0.0627***	0.9247***	0.0349***	0.0078***	4.5027
	(0.0337)	(0.0133)	(0.0009)	(0.0006)	(0.0012)	(0.0029)	
	*0.0000*	*0.0000*	*0.0000*	*0.0000*	*0.0000*	*0.0072*	
GED tails; volatility-based risk pricing	0.2215***	0.5131***	0.0659***	0.9214***	0.0303***	0.0000	
	(0.0322)	(0.0132)	(0.0008)	(0.0006)	(0.0012)	(0.0027)	1.1078
	*0.0000*	*0.0000*	*0.0000*	*0.0000*	*0.0000*	*0.9962*	
Gaussian tails; variance-based risk pricing	0.4917***	0.9738***	0.0691***	0.9176***	0.0220***	0.0007***	
	(0.0306)	(0.0048)	(0.0002)	(0.0001)	(0.0004)	(0.0001)	2.0000
	*0.0000*	*0.0000*	*0.0000*	*0.0000*	*0.0000*	*0.0000*	
Student’s t tails; variance-based risk pricing	0.4365***	0.4430***	0.0628***	0.9246***	0.0348***	0.0004***	4.5027
	(0.0337)	(0.0133)	(0.0009)	(0.0006)	(0.0012)	(0.0001)	
	*0.0000*	*0.0000*	*0.0000*	*0.0000*	*0.0000*	*0.0010*	
GED tails; variance-based risk pricing	0.2216***	0.5133***	0.0659***	0.9214***	0.0303***	0.0000	
	(0.0322)	(0.0132)	(0.0008)	(0.0006)	(0.0012)	(0.0109)	1.1078
	*0.0000*	*0.0000*	*0.0000*	*0.0000*	*0.0000*	*0.9374*	

**Notes:** Robust standard errors reported in parentheses and p-values presented in italics. Parameters ω, α, β, θ, μ, and ν represent unconditional variance, ARCH, GARCH, threshold, and conditional risk premia effects, and the distribution shape, respectively. *** denotes statistical significance at 1%.

**Fig. 3:**
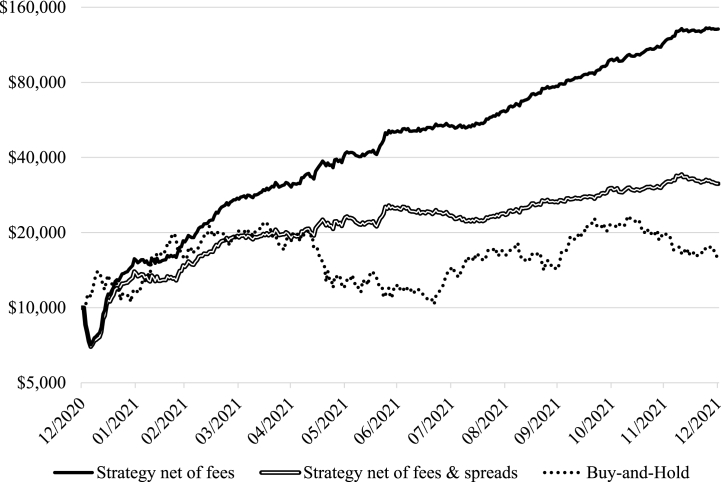
Strategy net performance on Bitfinex, 2021 (starting capital $10,000).

Next, this study considers a simulation of a high-frequency trading strategy seeking to exploit the turn-of-the-candle effect, which holds Bitcoin on Bitfinex in 2021 at the turns of the candle (0^th^, 15^th^, 30^th^, and 45^th^ minutes of each hour). Such a trading procedure is initially associated with prohibitively high fees, although a sufficiently large initial investment can allow an investor to achieve a zero fee 30-day volume threshold and eventusally outperform the buy-and-hold strategy. Table 5 below presents the results of a simulation without and with bid-ask spreads. Even when spreads are accounted for, a starting capital of as little as $5,000 generates 74.18% net per annum, exceeding 60.27% for the buy-and-hold. The strategy becomes implementable with a $3,585 initial investment, breaks even with $3,763, and matches the market performance with $4,725. Fig. 3 below also plots the equity curves for a hypothetical $10,000 investment (the minimum starting deposit on a Bitfinex account) and demonstrates its consistent outperformance.

**Table 5:** Turn-of-the-candle strategy performance for varying starting capital (Bitfinex, 2021).

Panel A: Net strategy performance after fees					
Capital	Time until zero fees, days	Breakeven time, days	Net return	Strategy cost	Maximum drawdown
$1,000	N/A	N/A	−100.00%	$1,000	100.00%
$2,000	N/A	N/A	−100.00%	$2,000	100.00%
$3,000	N/A	N/A	−100.00%	$3,000	100.00%
$3,402	N/A	N/A	−100.00%	$3,402	100.00%
$3,403	24	138	232.94%	$2,215	65.10%
$4,000	18	81	406.56%	$2,272	56.80%
$5,000	13	55	641.69%	$2,477	49.54%
$7,500	7	17	1011.91%	$2,768	36.91%
$10,000	5	12	1214.32%	$3,075	30.75%
$15,000	3	10	1425.25%	$3,557	23.71%
$25,000	2	8	1595.18%	$3,803	15.21%
$50,000	1	5	1723.23%	$4,404	8.81%
$100,000	0	3	1787.06%	$5,616	5.62%
Panel B: Net strategy performance after fees and bid-ask spreads					
$1,000	N/A	N/A	−100.00%	$1,000	100.00%
$2,000	N/A	N/A	−100.00%	$2,000	100.00%
$3,000	N/A	N/A	−100.00%	$3,000	100.00%
$3,584	N/A	N/A	−100.00%	$3,584	100.00%
$3,585	23	N/A	−12.86%	$2,348	65.48%
$3,763	21	333	0.00%	$2,360	62.71%
$4,000	18	263	16.32%	$2,346	58.64%
$4,725	14	91	60.27%	$2,538	53.71%
$5,000	13	78	74.18%	$2,589	51.78%
$7,500	7	18	164.59%	$2,938	39.17%
$10,000	5	13	213.88%	$3,180	31.80%
$15,000	3	11	265.13%	$3,726	24.84%
$25,000	2	10	305.97%	$4,108	16.43%
$50,000	1	5	336.81%	$5,042	10.08%
$100,000	0	4	352.10%	$6,936	6.94%

**Notes:** net return calculated using fees as per Bitfinex fee schedule, simulated rolling 30-day strategy volume, and Bitfinex-specific bid-ask spread retrieved from data.bitcoinity.org.

Further, to reinforce the abnormal return generating capacity of the strategy when both fees and spreads are accounted for, the probabilistic Sharpe ratio [4] for the strategy is computed and evaluated against the buy-and-hold strategy on daily data (see Table 6 below). The turn-of-the-candle exploiting strategy enjoys a Sharpe ratio of close to five, which is substantially higher than that of buy-and-hold (Z-statistic equals 9.78 and a probability of skill indicator is indistinguishable from 100%).

**Table 6:** Probabilistic Sharpe ratio evaluation of outperformance.

	Strategy net of fees & spreads	Buy-and-Hold
Return (annualised)	214.10%	62.95%
Risk (annualised)	42.95%	80.77%
Sharpe ratio	4.9635	0.7681
Skewness	0.0472	0.1490
Kurtosis	8.7168	1.6562
Standard error	0.4289	
Z-stat	9.7820	
Probability of skill	100.00%	

**Notes:** Calculated according to Bailey and Lopez de Prado [4] on daily returns.

**Fig. 4:**
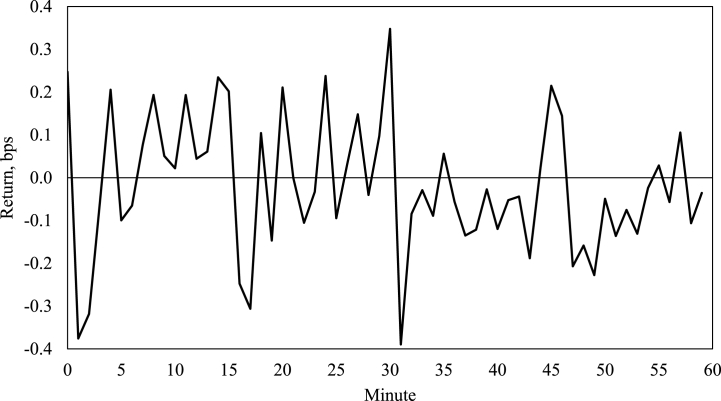
Turn-of-the-candle effect visualisation out-of-sample.

Finally, the turn-of-the-candle effect is computed for an out-ot-sample period comprising January through August 2022. Fig. 4 and Table 7 below present the results, demonstrating that, although less pronounced, the anomaly remains notable on the Bitcoin market after the cut-off point of the original sample this study utilised and therefore alleviating possible data-mining concerns.

**Table 7:** Out-of-sample test (Bitfinex, January–August 2022).

Model	OLS	Conditional median	Gaussian tails; volatility-based risk pricing	Student’s t tails; volatility-based risk pricing	Gaussian tails; variance-based risk pricing	Student’s t tails; variance-based risk pricing
Turn-of-the-candle (out-of-sample)	0.2916***	0.1126***	0.1810***	0.1127***	0.1809***	0.1127***
	(0.0745)	(0.0233)	(0.0304)	(0.0316)	(0.0304)	(0.0316)
	*0.0001*	*0.0000*	*0.0000*	*0.0004*	*0.0000*	*0.0004*

**Notes:** Robust standard errors reported in parentheses and p-values presented in italics. *** denotes statistical significance at 1%.

## 5 Conclusion

This study has established a recent, robust, statistically and economically significant anomaly on Bitcoin markets, with positive returns concentrated around the turn of 15-min candles, and average returns in all other trading minutes being negative. The results are consistent across exchanges, account for outliers, heavy tails, and conditional risk-pricing, and persist out-of-sample. A high-frequency trading strategy exploiting the “turn-of-the-candle effect” is net-profitable after fees and bid-ask spreads. Importantly, it outperforms buy-and-hold with an initial investment of as low as $5,000 and shows substantial informational content in the probabilistic Sharpe ratio framework.

An important venue for investigation in further studies is the conceptual origin of the anomaly. Empirical results are inconsistent with turn-of-the-candle being driven by blockchain fundamentals (as Bitcoin’s block time equals 10 min, not fifteen, and is very volatile block to block) or market sentiment (as the anomaly persists in bullish and bearish environments, most notably in 2022 for the out-of-sample test). The most plausible explanation can arguably be associated with the arrival of 15-min candle information itself and it being utilised by a variety of high-frequency trading bots and algorithms. This is somewhat consistent with the timing of the anomaly, as Petukhina et al. [26] demonstrated algorithmic trading was not very prominent on cryptocurrency markets as of 2018, and most exchanges have refined and expanded their API protocols since, which has undoubtedly been conducive to increasing trading bot activity. If such bots generate and trade upon signals that are noisy and optimistic enough, their reliance on 15-min candles (as well as 30-min and 1-h candles) could indeed be a compelling rationale of the turn-of-the-candle anomaly. Nevertheless, this remains a speculation and is ultimately left for future research endeavours on intraday cryptocurrency market dynamics.

This study has substantial implications for empirical finance cryptocurrency research, showing well-defined seasonal patterns exist in intraday Bitcoin returns and that novel inefficiencies have emerged in cryptocurrency markets recently. This is true for individual and institutional investors, demonstrating how a simple trading rule based on the turn-of-the-candle effect can generate material abnormal returns. Further research can investigate the persistence of the “turn-of-the-candle anomaly” as the market continues to evolve. Other cryptocurrencies should be subjected to the same analysis with the view on establishing concrete theoretical rationales for the origins of the anomaly.

## Author contribution statement

Savva Shanaev: Conceived and designed the analysis; Analyzed and interpreted the data; Wrote the paper.

Mikhail Vasenin: Conceived and designed the analysis; Contributed analysis tools or data; Wrote the paper.

Roman Stepanov: Conceived and designed the analysis; Wrote the paper.

## Funding statement

This research did not receive any specific grant from funding agencies in the public, commercial, or not-for-profit sectors.

## Data availability statement

Data will be made available on request.

## Declaration of interest’s statement

The authors declare no competing interests.
